# Correlation between maternal and umbilical cord blood in pregnant women of Pokhara Valley: a cross sectional study

**DOI:** 10.1186/s12884-018-1697-1

**Published:** 2018-03-21

**Authors:** Sameer Timilsina, Sirisa Karki, Aajeevan Gautam, Pujan Bhusal, Gita Paudel, Deepak Sharma

**Affiliations:** 1 Department of Physiology, Chitwan Medical College, Tribhuwan University, Post Box No, Bharatpur-13, Chitwan 42 Nepal; 2 Department of Pharmacology, Chitwan Medical College, Tribhuwan University, Bharatpur-13, Chitwan Nepal; 3 Department of Anatomy, Chitwan Medical College, Tribhuwan University, Bharatpur-13, Chitwan Nepal

**Keywords:** Hematology, Umbilical cord blood, Hemoglobin, Mother

## Abstract

**Background:**

Complete blood count is one of the routinely advised blood investigation during pregnancy. It is also utilized as a diagnostic tool for neonatal anemia, sepsis and determining hemostatic status of the newborn. The present study aims at estimating the complete blood count of maternal and umbilical cord blood at the time of delivery and to establish its correlation.

**Method:**

This cross sectional study included 114 mothers and their healthy neonates born through normal vaginal delivery. Complete blood count of umbilical cord blood and maternal blood was estimated using automatic hematology analyzer.

**Results:**

The mean maternal and neonatal hemoglobin concentration was 11.14 ± 1.39 g/dL and 16.34 ± 2.01 g/dL respectively. A significant positive correlation was found between maternal and fetal hemoglobin concentration (*p* < 0.001 and Pearson *R* = 0.496). The correlation between maternal and fetal WBC, RBC and Platelet count was not statistically significant. A significant positive correlation was found between maternal and fetal MCV and MCH while PCV showed a non-significant positive correlation.

**Conclusion:**

There was moderately positive correlation between maternal and fetal hemoglobin, MCV and MCH. The cord blood hemoglobin was lower in babies born to anemic mothers. The decrease in hemoglobin followed the severity of anemia, however, the correlation did not exist in anemic mothers. It suggested that fetal hematological parameters are not reflective of maternal hemogram.

## Background

Maternal physiology endures several alterations in hematological parameters with an average rise of 40–50% in blood and plasma volume during pregnancy [1]. This hemodilution produces pronounced effect in hematocrit values. Complete blood count is the most commonly advised blood investigation. It is often the first step in evaluating hematologic function and diagnosis of related diseases [2]. During embryonic period, hematopoiesis occurs in different parts of the developing fetus. It starts initially in the embryonic yolk sack migrating to the fetal liver and preterm marrow. However, hematopoiesis is primarily restricted to the bone marrow in adults [3].

Anemia is a clinical condition in which the number of circulating red blood cells is insufficient to meet the body’s physiologic demands. WHO classifies anemia [4] in pregnancy as non-anemic (> 11 g/dL), mild anemia (10–10.9 g/dL), moderate anemia (7–9.9 g/dL) and severe anemia (< 7 g/dL).

Umbilical cord blood hemoglobin is a dominant hematologic parameter of newborns at birth. Hemoglobin along with hematocrit values together have been customarily used in the diagnosis and follow-up of the neonatal anemia [5]. Besides this, other hematologic parameters like white blood cell count and platelet count are worthwhile in assessing neonatal sepsis and hemostatic status of infant [5]. Studies have shown some relationships between maternal and fetal hemogram but the extent of effect on maternal and neonatal health is still uncertain. This study was conducted to assess the effect of maternal hematologic profile during normal pregnancy on cord blood hematology.

In an attempt to achieving Millennium Development Goals (MDG) 4 and 5 Nepal has made noteworthy progress in the field of maternal, newborn and child health. Nepal Government has implemented certain health related policies like free ion supplementation and ANC visits even in the remote parts of the country. The policy also includes rewards for delivering babies in health care facilities. As a result, substantial improvement has been observed in maternal mortality rate plummeting from 450 in 2004 to 260 in 2014 [6]. As of yet, no studies have been conducted in Pokhara Valley of Nepal on maternal and neonatal hemogram. The present study aims at obtaining a complete picture of maternal and fetal hemogram thereby enabling to maintain referencing in the general population of this region. The anticipated advantage of this study was to determine the fetal outcomes beforehand and building up a strategy in order to prevent adverse outcomes.

## Methods

This was a hospital based cross-sectional study conducted from April 2014 to March 2015 at Manipal Teaching Hospital, Pokhara (827 m above sea level), Nepal. A total of 114 normal singleton pregnant mothers and their healthy newborn babies were included in the study. All pregnant women were residents of Pokhara Valley. They all have had 4 routine antenatal checkups during the course of pregnancy and all of them were supplemented with iron and folic acid. A written consent was obtained from the mother for participation in the study for both the mother and their newborn child. A brief history of the mother was taken during presentation for delivery. 3 ml of blood was collected in a sterile syringe from ante cubital vein and stored in an EDTA containing vial. After 2 min of delivery of the baby, 3 ml of umbilical cord blood was collected and was stored in an EDTA containing vial. Upon collection of the sample, it was analyzed within 3–6 h using an automatic hematology analyzer (Diagnova RFCL lablife h3d premier). Appropriate internal quality controls were run before assay of samples. Statistical analysis was performed by using the program Statistical Packages of Social Sciences (SPSS) version 20.0. The results were evaluated by using Pearson correlation test and Independent sample ‘t’ test. Shapiro Wilk test was done to check the normality of distribution of different variables. Multiple regression analysis was performed for multivariate analysis. Statistical significance was considered at *p* < 0.05.

## Results

The study included a total of 114 pregnant women and their newborns (86 males and 28 females). Of the total participants, 68(59.64%) were multi gravida and 46 (40.36%) were primigravida. The mean age of pregnancy was 26.04 ± 3.47 years with 86% (98/114) between the age of 21–30 years. The result presented here affirms the existence of variations in red cell indices of umbilical cord blood reported for Pokhara valley of Nepal. These results could be helpful in providing a baseline data for further studies as to establish a reference value for maternal and umbilical cord hemogram in the locality.

The result showed no significant correlation between total white blood cell count of mother with that of the fetus (*p* > 0.01). The normally distributed variables were hemoglobin, RBC count, hematocrit, MCV and platelets while the remaining variables were non-normally distributed.

**Fig. 1:**
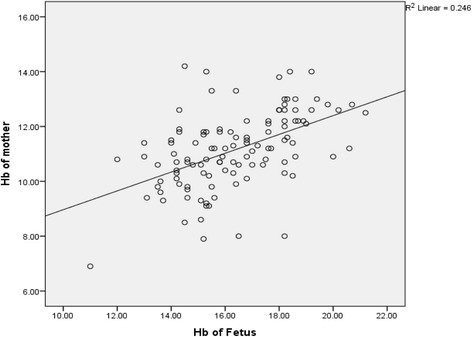
Scatter plot showing mean hemoglobin concentration of maternal and umbilical cord blood

**Fig. 2:**
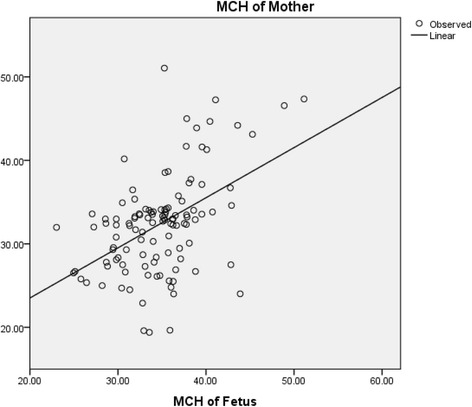
Scatter plot showing mean MCH concentration of maternal blood and umbilical cord blood

The mean maternal hemoglobin concentration was 11.14 ± 1.39 g/dL. According to the WHO classification of anemia in pregnant women, 46.49% of the participants were found to be anemic. The mean hemoglobin concentration of umbilical cord blood was found to be 16.34 ± 2.01 g/dL. The mean cord blood hemoglobin concentration in anemic and non-anemic mothers was 15.38 ± 1.7 g/dL and 17.21 ± 1.87 g/dL respectively. Figure 1 represents the scatter plot of mean hemoglobin of mother with that of the umbilical cord blood showing a linear correlation which was found to be statistically significant (*p* < 0.05). Likewise, Fig. 2 shows the scatter plot of mean MCH of mother with that of the umbilical cord blood indicating a linear correlation and was also found to be statistically significant (Tables 1 and 2).

**Table 1:** Mean Hemoglobin concentration of mothers in reference to classification of anemia by WHO.

Maternal Hemoglobin g/dL (WHO Classification)	Mean maternal Hemoglobin g/dL	No of patients	Pearson’s correlation coefficient R	*p* value
Non anemic Hb > 11 g/dL	12.17 ± 0.81	60	0.460	*p* < 0.05
Mild anemia Hb = 10–10.9 g/dL	10.57 ± 0.27	33	0.456	*p* < 0.05
Moderate Anemia Hb = 7–9.9 g/dL	9.18 ± 0.65	20	0.499	*p* < 0.05
Severe anemia Hb < 7 g/dL	……….	1	…….	………

**Table 2:** Correlation between maternal and fetal hemogram

Parameters	Mother	Fetal	Pearson’s correlation coefficient R	*p*-value
WBC (X 10^3^/mm3)	10.20 ± 3.36	15.52 ± 5.60	−0.040	NS
Hb (g/dL)	11.14 ± 1.39	16.34 ± 2.01	0.496	*p* < 0.001
RBC X 10^6^/mm^3^)	4.04 ± 0.46	4.72 ± 0.59	0.083	NS
Hematocrit (%)	33.29 ± 4.02	48.79 ± 6.88	0.109	NS
MCV (fL)	82.33 ± 6.46	102.85 ± 8.58	0.058	NS
MCH (pg)	33.34 ± 5.94	34.70 ± 4.73	0.478	*p* < 0.001
MCHC (g/dL)	33.40 ± 1.25	33.26 ± 1.09	−0.069	NS
Platelets (X 10^4^/mm3)	22.29 ± 6.61	229.20 ± 73.39	0.005	NS

Multivariate analysis showed maternal factors like age, gravida and smoking history did not influence the hematological parameters of the fetus. As all pregnant mothers were supplemented with iron and folic acid, ion supplementation was not considered as a confounding factor to umbilical cord hemogram.

## Discussion

The mean age of pregnancy was found to be 26.04 ± 3.47 years and the median age was 26 years which was slightly above the national data of census of Nepal 2011. WHO’s [4] report of 44% prevalence of anemia in Nepal was consistent with the present study. The result of the present study was homogenous to WHO survey for mean hemoglobin concentration of average Nepali pregnant women estimated at 11.1 g/dL (10.8–11.5 g/dL with 95% credibility interval) [4]. Similar to studies in other parts of the country, [7–10] 45.61% of pregnant women were found to have hemoglobin concentrations less than 10 g/dL and 0.8% with hemoglobin concentration less than 7 g/dL. This finding was far from similar to the 2016 Nepal DHS Key indicators [11] which reported anemia in this part of Nepal to be around 28%. The prevalence of anemia throughout the country is however similar to the present study (46%). [10].

The present study showed significant positive correlation between maternal and fetal hemoglobin concentrations like Dapper DV et al. [12] in Nigeria, McElroy PD et al. [13] in Kenya, and Alizadeh L et al. [14] in Iran. No significant correlation was observed between maternal and fetal white cell count and MCHC [12–14]. A positive linear Pearson correlation was observed between mean hemoglobin and PCV of cord blood and maternal blood. All these results were similar to the ones observed by Nneli R et al. [15] Singla PN et al. [16] and Al-hilli NM et al. [17]. In contrast to the present study, some previous investigators including Qaiser DH et al., [18] Kilbride J et al., [19] and Mamoury GH et al. [20] have failed to find a relationship between the maternal and cord blood hemoglobin.

The present study showed 5.7% of the fetuses had low hemoglobin concentration, 23.94% had low MCV, 18.24% had low MCH and none of the fetuses had low MCHC which was a constant finding with Abdelgader EA et al. [21] and Steer PJ [22]. The cord blood hemoglobin (16.8 g/dL) of healthy term neonates in this study was uniform with those reported from studies in the west [23, 24].

Qaiser DH et al. [25] measured the mean of all CBC parameters aiming to maintain refrencing, and found the mean hemoglobin = 14.99 ± 1.47 g/dL, RBC = 4.29 ± 0.44, PCV = 45.65 ± 4.83, MCV = 105.81 ± 6.24, MCH = 34.96 ± 2.11, MCHC = 32.47 ± 2.12, TC = 13.61 ± 4.23 and platelet count = 256.25 ± 76.54 in the umbilical cord blood. The result showed similar findings with the present study but with a slightly higher total WBC count.

As the study was conducted in only one tertiary care center and that too with a limited number of patients, the result does not represent the pregnant women in Western Development Region of Nepal. Also, the study only included the patients attending hospital for delivery so the results cannot be generalized as it may not be a true reflection of the general population. However researchers have found the increasing trend of hospital based deliveries in urban population and Pokhara being an urban city, the data and results presented could actually represent the urban population of Pokhara valley. Socioeconomic status of the pregnant women was also not considered in the study. Moreover, further studies are to be conducted in the field to achieve complete results.

## Conclusions

There was moderately positive correlation between maternal and fetal hemoglobin, MCV and MCH. There was a decrease in the cord blood hemoglobin in different severities of anemia, however, the correlation between maternal and fetal hemoglobin did not exist. It suggested that the fetal hematological parameters are not reflective of maternal hemogram.

Anemia in pregnancy should be properly evaluated and treated accordingly in order to avoid unwanted fetal and maternal outcomes.

## Abbreviations

ANC: Ante-natal Checkup
CBC: Complete Blood Count
EDTA: Ethylene Di-amine Tetra Acetic acid
Hb: Hemoglobin
MCH: Mean Corpuscular Hemoglobin
MCHC: Mean Corpuscular Hemoglobin Concentration
MCV: Mean Corpuscular Volume
MDG: Millennium Development Goals
PCV: Packed Cell Volume
RBC: Red Blood Cells
SPSS: Statistical Package for Social Sciences
TC: Total Count
WBC: White Blood Cells
WHO: World Health Organization
